# Gut Microbiome and Depression: How Microbes Affect the Way We Think

**DOI:** 10.7759/cureus.9966

**Published:** 2020-08-23

**Authors:** Therese Limbana, Farah Khan, Noha Eskander

**Affiliations:** 1 Psychiatry, California Institute of Behavioral Neurosciences and Psychology, Fairfield, USA

**Keywords:** gastrointestinal microbiome, depression, gut microbiome, gastrointestinal microbiome and depression, gut microflora, bacterial commensals, gut microbiome and mental health, intestinal microbiota, intestinal microbiota and depression, bacterial commensals and depression

## Abstract

The gut microbiome serves an important role in the human body. Reportedly, one of the benefits of these microflora is on mental health. Once established, food and other dietary sources that enhance quality microbiome content in our gastrointestinal system will be a significant consideration in individuals’ day to day lives. This literature review conducted a PubMed search for studies about the gut microbiome and its relation to depression. In using several Medical Subject Heading (MeSH) keywords, relevant literature was selected. A total of 26 articles were selected after applying the inclusion and exclusion criteria, and after checking the articles’ accessibility. This literature would like to establish the role of the gut microbiome in depression. This study's findings showed that there is a strong association of microbiome function to mental well-being.

## Introduction and background

Microbes in the gut are of great importance to the human body. The composition of one's gut microbiota is individually specific and is highly influenced by genetics, growth and development, and location [1]. With an estimated 1018 microorganisms, mostly made up of anaerobic bacteria, the gut microbiome is responsible for multiple functions in bowel movement, digestion of food, and absorption of nutrients [2]. With the brain and the gut working in a bi-directional manner, they could affect each other's functions and significantly impact stress, anxiety, depression, and cognition [3].

Depression is a serious mental illness caused by multiple factors [4]. It is described as low emotional disposition, loss of confidence, and apathy [2]. Depression is suggested to result from complex interactions of an individual's genetics and their environment. Major depressive disorder (MDD) tops the spot in contributing to the worldwide disease burden, as claimed by the World Health Organization (WHO) [5]. Based on the WHO reports, there are approximately 350 million people affected by depression [6]. Research findings showed that healthy gut microflora transmits brain signals through the pathways involved in neurogenesis, neural transmission, microglial activation, and behavioral control under stable or stressful conditions. This process led several studies to recognize the importance of microbiomes in managing mental health issues [7]. In depression, there is also dysregulation of the neuroendocrine and neuroimmune pathways [8,9]. More than 20% of Inflammatory Bowel Disease (IBD) patients have sleep disturbances and depressed behaviors. By acknowledging that inflammation affects the brain and how one thinks, treatments addressing this phenomenon have grown its popularity in IBD patients and healthy populations [10].

The study of gut microbiota affecting mental health is a relatively new research topic that has gained popularity these past years. There are still parts that need to be delved deeper and to be understood. A comprehensive evaluation of the gut microbiome leading to depression reveals flaws in the research design and how it was performed, suggesting that results may be subpar compared to other research studies [11]. Furthermore, more studies are needed to ascertain the benefits of using probiotic interventions in promoting stable brain processing [3]. For example, the laboratory findings in rodent studies have not yet been clear on the effects of these gut microbiota in modulating psychiatric illnesses [12]. The degree of changes in function and composition of gastrointestinal microflora leading to depression [9] and the causal connection of both the bacterial commensals and depression have to be fully understood to establish the role of the gut microbiome in depression [13]. The environmental factors contributing to MDD are also still unclear [5]. With all the findings noted in the research of gut microbiome and depression, this literature review aims to establish an association between gut microbiota and depression and how these gut microbes affect mental health.

## Review

Method

A literature search was done through the use of MeSH keywords in PubMed. Table 1 indicates the keywords used in a literature search. 

**Table 1:** Medical Subject Heading (MeSH) keywords used for the literature search

MeSH Keywords:	
Gastrointestinal Microbiome (Subheading- Etiology)	
Total Records	2984
Records Selected	2005
Gastrointestinal Microbiome (Subheading- Physiology)	
Total Records	5532
Records Selected	3772
Depression (Subheading- Anatomy, and Histology)	
Total Records	1608
Records Selected	1072
Depression (Subheading- Etiology)	
Total Records	34581
Records Selected	28883
Depression (Subheading- Psychology)	
Total Records	36971
Records Selected	33984
Gastrointestinal Microbiome (Subheading- Etiology, and Physiology) and Depression (Subheading- Anatomy and Histology, Etiology, and Psychology)	
Total Records	44
Records Selected	27

The study's inclusion criteria include the use of human studies, published papers in the English language, and all the abstracts and full papers. On the other hand, the exclusion criteria were fully non-human studies and non-English language.

Results

Table 2 presents the total number of articles based on the inclusion and exclusion criteria.

**Table 2:** The total number of articles based on the inclusion and exclusion criteria MeSH: Medical Subject Heading

MeSH Keywords:	
Gastrointestinal Microbiome (Subheading- Etiology)	
Total Records	2984
Human Population	2047
English Literature	2938
Records Selected	2005
Gastrointestinal Microbiome (Subheading- Physiology)	
Total Records	5532
Human Population	3889
English Literature	5405
Records Selected	3772
Depression (Subheading- Anatomy, and Histology)	
Total Records	1608
Human Population	1138
English Literature	1530
Records Selected	1072
Depression (Subheading- Etiology)	
Total Records	34581
Human Population	32220
English Literature	31113
Records Selected	28883
Depression (Subheading- Psychology)	
Total Records	36971
Human Population	35823
English Literature	35088
Records Selected	33984
Gastrointestinal Microbiome (Subheading- Etiology, and Physiology) and Depression (Subheading- Anatomy and Histology, Etiology, and Psychology)	
Total Records	44
Human Population	28
English Literature	43
Records Selected	27

After doing a refined search using the MeSH keywords 'Gastrointestinal Microbiome (Subheading- Etiology, and Physiology)' and 'Depression (Subheading- Anatomy and Histology, Etiology, and Psychology),' 27 articles were retrieved. Out of the 27 articles, one was excluded because of the unavailability of abstract and inability to access the paid article. 

**Figure 1:**
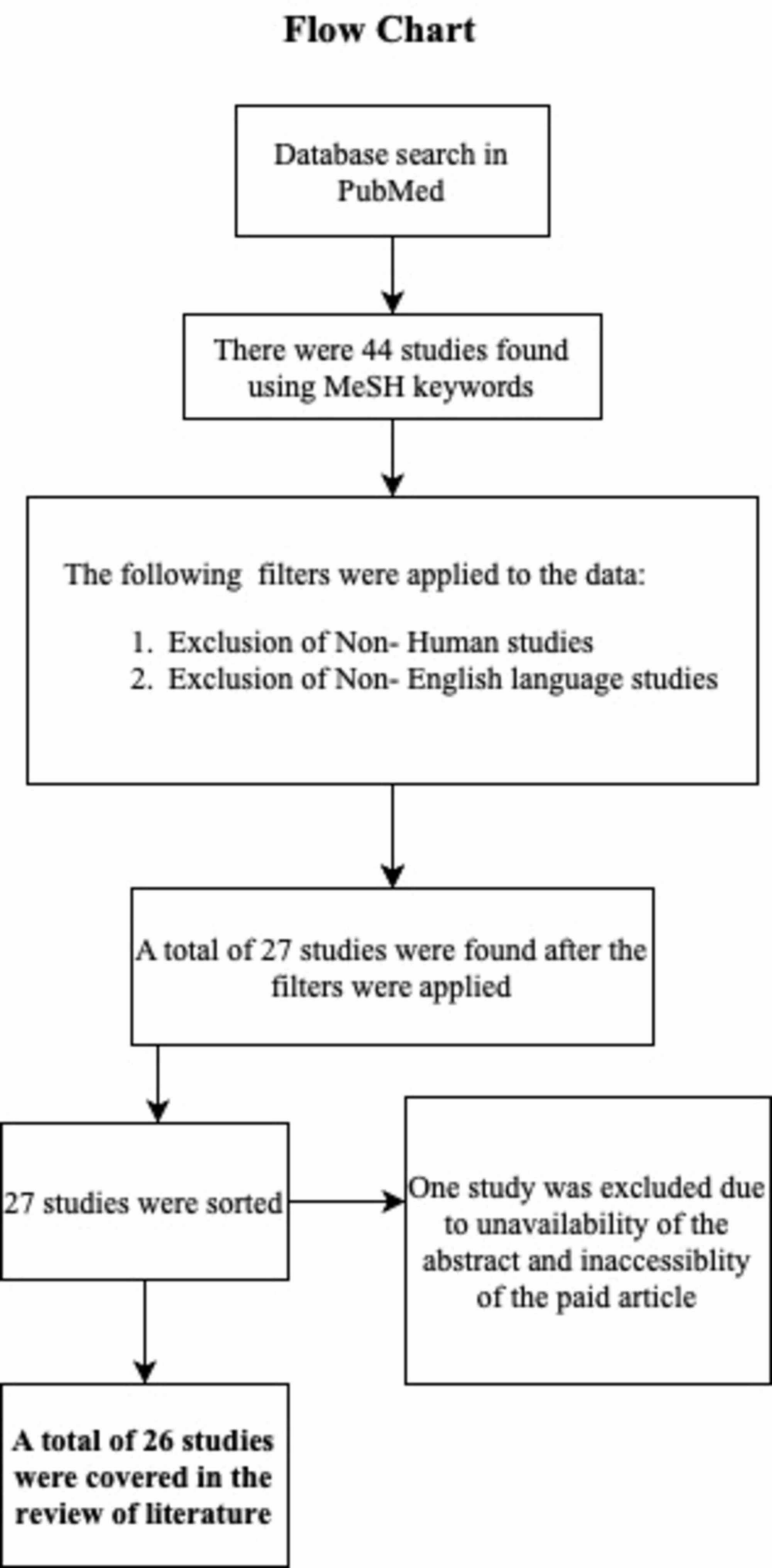
Flow chart of the literature review process

Figure 1 below exhibits the process of the current literature review.

Discussion

*Overview* 

There are approximately 100 trillion microbes from a thousand diverse species residing in the human gut. Each microflora serves a specific role and is affected by food choices, genes, medication exposures, and stressful events [1]. Gut microbiome or intestinal microflora serves several purposes in the body. They affect the promotion of gastrointestinal digestion, food absorption, and maintenance of intestinal integrity. In the past few years, the relationship of gut dysbiosis to disease conditions are established. There is a bidirectional relationship between the two entities, and studies noticed a specific microbe for every disease developed, especially the illnesses in mental health [2]. There are several pathways implicated in the link. Several of the pathways are neuroimmune, neuroendocrine, and sensory-neural. Although a connection is noted between microflora leading to depression, there is still a need to establish the causality link by analyzing the role of each gut microbiota- the chronology of events that comes first is yet being studied. In the case of rodents, depression developed in fecal transplant recipients after receiving fecal samples from MDD patients. However, rodents who have experienced stress and depression showed a reduction in gut microbiome content and diversity [13].

Gut Microbiome and Depression

Any permutations in the gut microbiome composition trigger microbial lipopolysaccharides (LPS) production. It, in turn, activates inflammatory responses. Cytokines send signals to the vagus nerve, which links the process to the hypothalamic-pituitary-adrenal axis that consequently causes behavioral effects. Another school of thought suggests that the gastrointestinal (GI) tract's inflammation leads to neuroinflammation. It then fuels microglial action and triggers the kynurenine pathway. All these processes induce depression [14]. In human studies, evidence of changes in microflora composition explains depression [8]. The bidirectional connection between gut microflora and depression has been well reinforced by research. But as mentioned, the direction of causality between the two entities still needs to be determined [13]. Many research studies have directed their attention to understanding the interaction between the gut microbiome and the brain. The use of supplemental probiotics has shown promising effects on addressing brain-associated problems. Although encouraging, more research is needed to ascertain probiotics' mode of action and side-effects [3]. Under stable and stressful conditions, the gut commensals may send signals to the central nervous system (CNS) through different pathways such as neurogenesis, neurotransmission, and many more. The potential of stressful events to control our gut microbiome content and the ability of these bacterial commensals to manage stress levels have been uncovered.

Patients diagnosed with mental conditions, including depression, have demonstrated gut microbiome dysbiosis [7]. Intricate relationships of the individual gene and the surroundings are pointed out to contribute to the development of MDD. The lack of bacterial microflora in germ-free (GF) mice reduced the immobile period in the forced swimming test compared to the healthy mice. The composition and diversity of bacteria between healthy and depressed patients showed significant disparity with the depressed group showing mostly Firmicutes, Bacteroides, and Actinobacteria in the gut. This supports how the gut bacterial content's dysbiosis changes the behavior of the host [5]. In recent years, neurobiological modifications have been related to the development of depression. Inflammation is one of the connections that leads to the modification process. It showed high levels of concentration of neurotransmitters in inflammation, and increasing psychological processes are noted. In maintaining the regulation of physiology, the host must keep a stable microbial community safely to say that these microbiomes have control in curbing depression [6]. Several factors also send signals to the brain regarding the gut state, such as infectious substances, cytokines, antibiotics, and vagal sensory fibers, to name a few. The hypothalamic-pituitary-adrenal axis also metabolizes microbiome diversity and nutrient ability [15].

Inflammation to Depression

Studies have shown that patients with inflammatory diseases are prone to depression. Worsening IBD conditions are anticipated by more frequent bouts of depressed symptoms. The theory is that dysregulation of the pathways involved in the gut-brain axis is linked to this phenomenon [10]. In understanding the interplay of inflammation leading to depression, and depression worsening cytokine responses, it is essential to fully understand the process to stop this vicious cycle [16]. New research findings reveal that prolonged neuroinflammation affects brain functions, which could dictate the individual's mood and behavior [4]. The interaction of both depression and inflammation is like a vicious cycle fueling each other. Inflammation is one of the leading forces in the development of depression. It consequently triggers more cytokine production in response to the stressors that are not innate to the human body. There is an amplified inflammatory process when several factors, including genetic risks, pathogens, and environmental stressors, co-occur. The effects noted like depression and other harmful practices such as poor diet choices and lack of exercise may hasten the inflammatory reaction uncontrollably and worsen depression. Any stressful events experienced by an individual dictates the diversity and composition of their gut commensals. The recurrent, immense, and chronic inflammation worsens mental and physical conditions. When both inflammation and depression are presented in a patient, it is essential to provide management for both issues to resolve the problem [17].

The prevalence of depression in IBD patients has been increasing, which worsens the affected groups’ quality of life. Both conditions involve immune-inflammatory, oxidative (IO), and nitrosative stress (NS) pathways. These are exhibited by increasing levels of pro-inflammatory cytokines and acute-phase reactants as well as decreasing levels of negative acute phase reactant and many more. The IO and NS processes of both depression and IBD overlap, insinuating a causal relationship. These processes have vital importance in the management of either condition [17].

Diet

Certain types of diet are associated with improving mental health. One example is the Mediterranean diet, which promotes healthy eating, unlike the Western counterparts. Diet choices significantly affect other body systems, such as the endocrine, immune and gastrointestinal systems [18]. One study found that Western diets contribute to the dysbiotic conditions that send brain signals to alter diet intake behavior [19]. High-fat consumption not only leads to obesity but also causes widespread inflammation of body systems. The gut microbiome may alter the harmful effects of the high-fat diet, improving mood and behavior. By modulating gut microbiome composition through proper nutrition and probiotics, we also help decrease anxiety and depression [4]. Lactobacilli and inflammation are also recognized to affect the brain pathway and when an imbalance occurs, mood disorders develop [20].

We are still in the early period of research into how diet can influence our brain processes. A large-scale clinical trial conducted did not show significant effects of the Mediterranean diet on the behavior of adults with subclinical manifestations of depression. However, when looking at the smaller-scale clinical trials, improvement of depressed symptoms was noted after shifting the current diet to the Mediterranean. These trials also considered the subjects' expectations and how dietary changes have improved their overall perception of their well-being [18]. Although studies regarding this phenomenon are conducted to depressed human populations and rodent models, the evidence proving that gut microflora dysbiosis is the main causative factor is still inadequate. Clinical parameters to measure gut microbiome alteration are yet to be established [19].

Marital Potential to Develop Diseases

Stressful times push individuals to engage in unhealthy behaviors, such as lack of sleep and unhealthy food choices. These practices lead to increasing gut leakiness that promotes inflammation and immunosenescence. These risks are modified through reverting to healthy lifestyle practices by proper diet, exercise, and adequate sleep [21]. In married couples, it was found that they do not just affect each partner’s physical health but also mental health. The different life stressors, challenges, and disturbance in their shared lifestyles influence their health condition. They are promoting some disease risks, which may also affect their gut microbiota homeostasis. Exposure to chronic relationship problems increased the chance to develop metabolic syndrome and cardiovascular issues through their bodies' production of insulin and peaked triglyceride levels after poor quality dietary intake [21].

Several studies strongly support the association of gut microbiome in the development of depression. Table 3 shows some of the studies that link the gut commensals and their effect on the hosts' mental health.

**Table 3:** Some of the studies with a positive association of gut microbiome to depression CRP- C- Reactive Protein; ARNTL- Aryl hydrocarbon Receptor Nuclear Translocator-Like; CPG- 5'-C-phosphate-G-3'; LBP- Lipopolysaccharide-Binding Protein; CD14- Cluster of Differentiation 14; MDD- Major Depressive Disorder

Author/Publication Date	Study Design	Sample size	Main Points
Heym et al. [20], 2019	Observational Study	40	There is a bi-directional link between gut bacterial commensals and the central nervous system; the study involved 40 subjects; fecal and blood samples were collected to assay the microbiota as well as pro-inflammatory molecules; hierarchical regression analyses reveal limited cognitive empathy and poor self-judgment predicted depression; the more diverse gut microbiota led to positive self-judgment and protection from depression; the level of CRP predicts the negative cognitive empathy
Bengesser et al. [22], 2019	Observational Study	32	The gut microbiota contains more genetic material than the cells of our entire body; it has direct effects on the different physiological process and creates a link to the brain through the gut-brain axis; the study intends to establish the correlation between the gut microbiome diversity and the clock gene ARNTL gene methylation in Bipolar Disorder patients; ARNTL CPG position methylation is linked to diversity and evenness; there is a significant difference of bacterial composition between depressed patients and patients on euthymic episodes; gut bacterial microflora diversity is inversely correlated to the ARNTL methylation
Kiecolt-Glaser et al. [23], 2018	Clinical trial	86	Depression and marital stressors potentiate the risk of developing inflammatory conditions; bacterial translocation promotes systemic inflammatory responses; the study involves a secondary analysis of a double-blind experiment assessing lipopolysaccharide-binding protein (LBP) and soluble CD14 (sCD14); there were 43 healthy married subjects within the age range of 24-61 who were asked to talk about relationship conflicts on two occasions; it showed that negative marital behaviors lead to the distress of both partners; the study reveals that aggressive interactions between the couples led to a higher LBP level than the less hostile group; when mood disorder history was added, higher LBP/sCD14 ratio was noted; stressful marriage and history of mood disorders promote inflammatory responses that worsen the condition
Kelly et al. [9], 2016	Observational/ Laboratory Study	67	Multiple pathways were noted linking both gut microbiota and the nervous system; there is a bidirectional relationship affecting behavior and brain functions; there were 34 subjects with major depressive disorder (MDD) and 33 healthy subjects as the control group; blood and fecal samples were collected; fecal microbiota sample came from MDD patients and was transplanted to rats with depleted gut microflora; depressed behaviors developed in the microbiota transplanted rats

Although numerous studies have been conducted about the gut microbiome and its strong effect on mental health, there are still studies that negate these findings and suggest conducting more research experiments and better methods in finding out more details. Table 4 contains some of the reviews not supportive of the connection between the gut microbiome and depression. 

**Table 4:** Some of the studies with a negative association of gut microbiome to depression BMI- Body Mass Index; rRNA- Ribosomal Ribonucleic Acid

Author/Publication Date	Study Design	Sample size	Main Points
Kleiman et al. [24], 2017	Observational Study	91	The study measured the gut microbiome content and correlated the findings to the host's mental status; there were 91 females between the ages of 19-50 years and. Within the BMI of 18.5-25 kg/m^2 ^as subjects of the study; each participant provided fecal samples and answered psychiatric questionnaires; 16s Illumina Sequencing of rRNA gene measured bacterial content; result associations were studied through the use of Rendall’s Tau-b Correlation Coefficient with Benjamini and Hochberg's False Discovery rate procedure; the result showed no associations between microbiome content and psychiatric questionnaire scores; the findings did not support the role of the gut-brain axis in homeostatic conditions responding to psychiatric health measures; the role of gut microflora may be specific only to worse mental conditions
Kelly et al. [12], 2016	Literature review		The function of bacterial microflora is still not fully clarified in groups with psychiatric illnesses. Although enticing to theorize that patients may receive help from using the gut microbiome in managing mental health problems, reliable results are still yet to be proven in rodent studies.

This literature review also found the importance of an individual's resilience in maintaining one's immunity. The immune system serves as one of the main body systems affected by the gut microbiome to the depression pathway. Resilience is the ability to endure and recover from adverse events encountered by the individual. Research findings showed that the qualities that assist in fostering resilience have protective effects in combatting the stressors affecting other systems of the body. More data were retrieved, indicating that the resilient group of people has a unique immunophenotype compared to their stress-vulnerable counterparts. However, it was found that this stress-vulnerable group can be converted to resilient individuals or vice-versa by manipulating their immunophenotype. The resilient inflammatory phenotype also tenders an ability to bounce back better from inflammatory symptoms [25]. Although the link of gut microbiota to mental health is growing in popularity to the research world, more studies are needed to prove the relationship between resilience and immunity [25].

After gaining knowledge about how microbiomes affect human behavior, giving importance to the food we eat, refraining from taking those medications with an adverse impact on our gut commensals, and considering probiotic supplements part of our diet regimen is essential [14]. The relevance of gut microbiota to the body remains unpopular to society, albeit already relevant to the research community. In the quest for reducing IBD exacerbations, depressive behaviors are also targeted by providing anti-inflammatory management [10]. It will be a medical breakthrough once the inflammatory pathway is proven to be a part of the pathophysiology of psychiatric diseases. More clinicians will be aware that stressful events encountered or the genes inherited are not the only contributors to the mental condition, but also the inflammatory process experienced by the patient. Effective psychiatric illness management would mean that we address both the behavioral aspect of illness and the inflammatory process experienced by the patient, leading to overall health [16]. Gut and neural pathways are other aspects implicated in the development of depression after exposure to traumatic events in early life, early drug intake destroying the gut commensals, and poor childhood nutrition choices. This information is an essential guide for taking care of the pediatric population [6]. 

A good understanding of the bacterial commensals’ mechanism may lead to microbiome-based breakthrough treatments [7]. It is recommended that more laboratory investigations be performed to establish the microbiota-gut-brain (MGB) axis functions [26]. Although the MGB axis is slowly gaining in popularity, there are still limited research studies on the topic. Complete understanding of the link of the MGB axis and neuroimmune systems directs the future to more successful depression management [15]. More laboratory studies are highly encouraged to be conducted focusing on ascertaining the details on specific concentration levels of gut microbiota in the development of specific mental health illnesses. Also, more large-scale studies are needed to strengthen the results of earlier research studies. The present literature review has limitations that should be taken into consideration. The study restricts its scope in terms of language (English language studies only), and only human studies were covered. All types of research designs were welcomed in this literature review.

## Conclusions

This study intends to determine the role of the gut microbiome in mental health and depression. This literature review established a strong link of microbes in the gastrointestinal tract, affecting how individuals think and how the gut-brain axis serves as an essential pathway in considering the management of several mental issues and psychiatric illnesses. Although promising, gut microbiome studies have a long way to go. More laboratory experiments need to be proven regarding the composition, qualities, or concentrations of gut microorganisms dictating certain behaviors within the spectrum of mental health and illness. We are still far from establishing the role of these fantastic gut microbes to how we think. Nevertheless, once we understand the specifics fully, we will be able to break the hindrances in the management of many intractable psychiatric diseases.
